# A “virtually minimal” visuo-haptic training of attention in severe traumatic brain injury

**DOI:** 10.1186/1743-0003-10-92

**Published:** 2013-08-09

**Authors:** Assaf Y Dvorkin, Milan Ramaiya, Eric B Larson, Felise S Zollman, Nancy Hsu, Sonia Pacini, Amit Shah, James L Patton

**Affiliations:** 1 Rehabilitation Institute of Chicago, 345 E. Superior Street, Chicago, IL 60611, USA; 2 Department of Physical Medicine and Rehabilitation, Northwestern University, Chicago, IL, USA; 3 University of Illinois at Chicago, Chicago, IL, USA

**Keywords:** Virtual reality, Robotics, Attention, Rehabilitation, TBI

## Abstract

**Background:**

Although common during the early stages of recovery from severe traumatic brain injury (TBI), attention deficits have been scarcely investigated. Encouraging evidence suggests beneficial effects of attention training in more chronic and higher functioning patients. Interactive technology may provide new opportunities for rehabilitation in inpatients who are earlier in their recovery.

**Methods:**

We designed a “virtually minimal” approach using robot-rendered haptics in a virtual environment to train severely injured inpatients in the early stages of recovery to sustain attention to a visuo-motor task. 21 inpatients with severe TBI completed repetitive reaching toward targets that were both seen and felt. Patients were tested over two consecutive days, experiencing 3 conditions (no haptic feedback, a break-through force, and haptic nudge) in 12 successive, 4-minute blocks.

**Results:**

The interactive visuo-haptic environments were well-tolerated and engaging. Patients typically remained attentive to the task. However, patients exhibited attention loss both before (prolonged initiation) and during (pauses during motion) a movement. Compared to no haptic feedback, patients benefited from haptic nudge cues but not break-through forces. As training progressed, patients increased the number of targets acquired and spontaneously improved from one day to the next.

**Conclusions:**

Interactive visuo-haptic environments could be beneficial for attention training for severe TBI patients in the early stages of recovery and warrants further and more prolonged clinical testing.

## Background

Traumatic brain injury (TBI) affects 1.5 million people each year in the United States alone and frequently leads to a variety of sensorimotor and cognitive deficits [1]. Among these, attention deficits are one of the most profound problems facing the traumatic brain injured individual [2]. Inattentiveness and difficulty focusing and concentrating on a task are among the most prominent symptoms. Survivors of moderate-to-severe TBI, especially in the acute and subacute phases, exhibit deficits in the more basic aspects of attention. Later in the recovery process patients exhibit more subtle deficits. Since attention plays a major role in many cognitive functions, attention has been the target of various types of rehabilitation programs for TBI survivors, for both inpatient rehabilitation and postacute or community re-entry settings [3,4]. However, treatment outcome studies for the inpatient population, especially the severely impaired population, are scarce [3,5-7]. There have been even fewer studies that might advance new treatments that can be tolerated by this population.

The most studied approach to attention rehabilitation is the Attention Process Training (APT), which provides the patient with repetitive attention exercises that increase in complexity [8]. Several studies that evaluated the effectiveness of APT, demonstrated the beneficial effects of APT on attention [9-13]. Other novel approaches to attention rehabilitation teach compensatory strategies [14] and use computers to remediate attention [15,16]. Evidence-based reviews of the various studies show that while rehabilitation programs tend to improve attention in the more chronic and higher functioning patients, to date, there is insufficient evidence for the effectiveness of specific interventions for attention deficits for the inpatient population at the early stages of recovery [4,17,18].

Technological advancement in robotics and display technology in recent years has enhanced our ability to provide new rehabilitation pathways. With new technology in robot-rendered haptics (sense of touch), several rehabilitation studies have demonstrated great promise. Virtual Reality (VR) can be used to create relevant simulated environments with which a user can interact and where treatment of cognitive and motor deficits can take place. It provides highly controllable interactive environments that support repetitive delivery. It also provides the ability to introduce distractions when required, objectively measure, and remediate attention in challenging, safe, and meaningful environments. Robotic devices can be integrated with a VR system to allow more sophisticated visuomotor interactions that can also quantify various aspects of cognitive and motor functions [19].

VR and robotics technology have been shown to be an effective tool in different domains of therapy in TBI [20-24], and have been shown to enhance patient motivation and enjoyment [25,26], important factors in successful rehabilitation [27]. The majority of studies testing the efficacy of using these technologies in rehabilitation however were done with the stroke population. To date, there is a paucity of literature on the use of technology that stimulates visuomotor interactions in the TBI population, especially for assessment and rehabilitation of attention. This is ironic, because physically salient stimuli have been known to capture attention. Previous studies have demonstrated that sustained spatial attention operates across sensory modalities such as vision and touch [28,29]. It has also been shown that spatial information from tactile cues is effective at directing overt visual attention to locations in space [30]. Moreover, studies have shown that integrating visual and tactile stimuli results in better performance compared with individual presentations in either modality alone [31-33]. Therefore, integrating visual targets with haptic cues such as nudges during attention loss (a pause in movement) or a haptic break-through barrier around the visual target (a resistive haptic force giving subjects a “break-through” sensation as they acquired the target) might better recapture or sustain attention in subjects who have experienced a brain injury. In a previous preliminary study with fewer subjects, we demonstrated that such haptic interactions were well-tolerated by moderate-to-severe TBI survivors (Rancho Los Amigos level of IV-VII; [34]) who received acute inpatient rehabilitation [24,35]. The study further analyzed the efficacy of an application that provided consistent cues when participants exhibited off-task behavior [24].

Here we expand on our previous work on a larger sample size of 21 severely impaired TBI inpatients (Rancho level of IV-V), to evaluate how haptic cues might be beneficial for remediation of attention, and we further analyze the spatial and temporal kinematics of movements. The severely impaired TBI inpatient population during early stages of recovery is challenging. It was therefore important to evaluate the parameters necessary for a future protracted study with repeated treatments. It was hypothesized that a technology that allows control of environmental distractions and of task difficulty might lead to short-term benefit (increase number of targets acquired), which in turn might lead to longer-term benefits (increase attention). It was also hypothesized that haptic feedback would capture, redirect and help sustain the patient’s attention, and a target that could be both seen and felt would enhance performance by increasing number of targets acquired. The main findings revealed that this group of patients improved over time and benefited from haptic cues while showing some degradation of performance when face with haptic break-through barriers.

## Methods

### Subjects

21 severe TBI patients (17 males, 4 females) in the early stages of recovery were recruited from the inpatient unit at the Rehabilitation Institute of Chicago. Personal and clinical details are shown in Table 1. Patients who were recruited for the study had attention deficits, considered to be in early stages of recovery (scoring IV or V on the Rancho Los Amigos scale, based on clinical evaluation pre-test), and had upper extremity strength of at least 4 out of 5. Patients had no visual field defects or hemispatial neglect that prevented perception of test stimuli. All procedures were approved by the Institutional Review Board of Northwestern University.

**Table 1:** Personal and clinical details

**Gender**	**Age**	**Weeks since injury**	**RLA (level IV and V)**
17 M; 4 F	37.8 ± 17.9	10.3 ± 15.6	5 IV; 16 V

*RLA* Rancho Los Amigos levels of cognitive functioning scale.

*M* Male.

*F* Female.

### Apparatus and data collection

**Figure 1:**
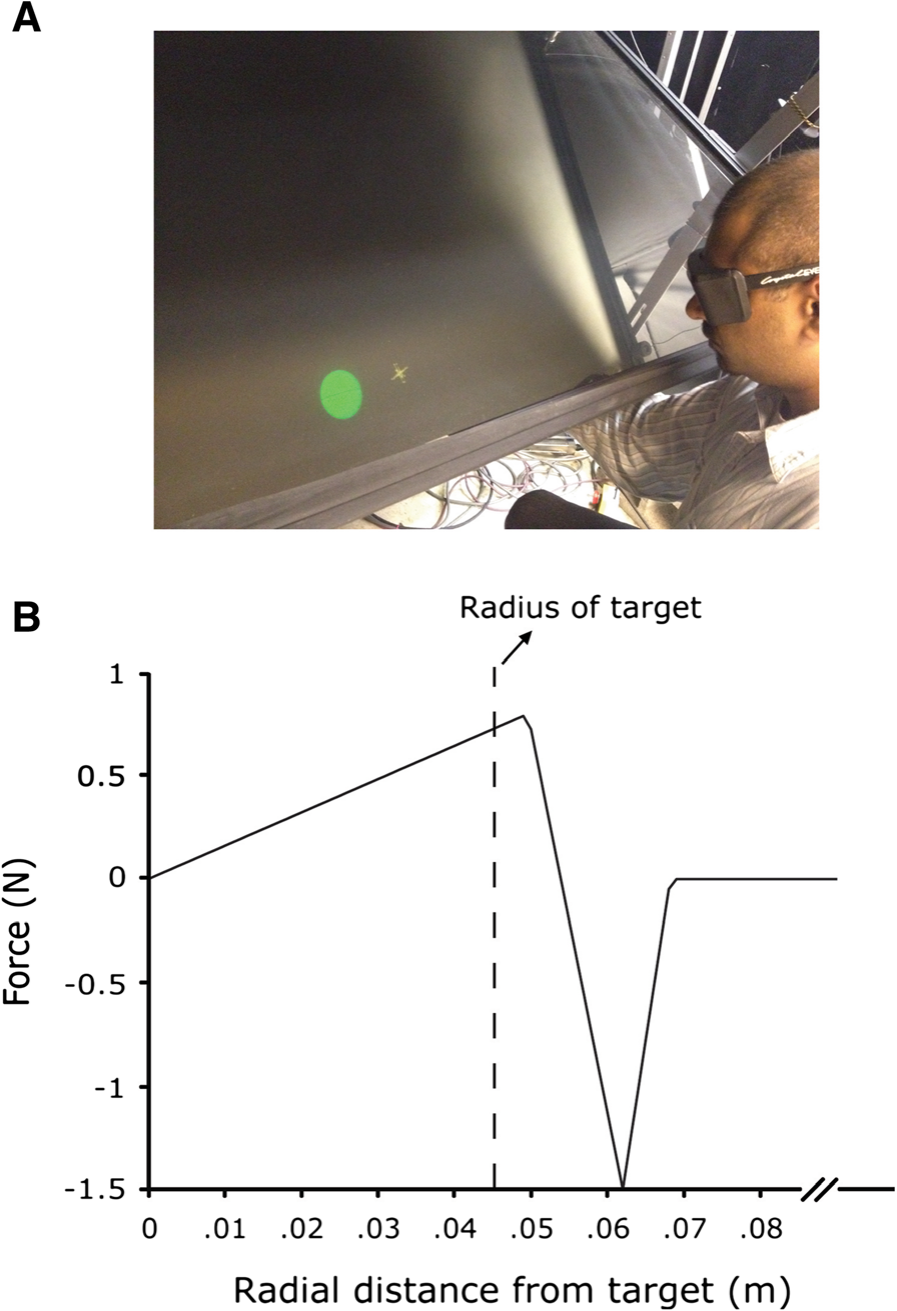
**Apparatus and design of break-through force. (A)** Screen shot of an individual performing within the VRROOM system. **(B)** The change in force as a function of distance from the target center for the break-through condition.

The VRROOM (Virtual Reality and Robotics Optical Operations Machine), a three-dimensional haptics/graphics system was used for this study. A cinema-quality digital projector (Christie Mirage 3000 DLP) displayed the images over five-foot-wide 1280x1024 pixel image resulting in a 110° wide viewing angle [19]. A 6-degree of freedom PHANToM Premium 3.0 robot (SensAble Technologies) was used to measure arm movement and apply forces during movement. The correct perspective and stereo projections for the view of the scene were computed using values for the current position and orientation of the head (6 DOF) supplied at 100 Hz by a tracking sensor (‘Flock of birds’, Ascension Technology) attached to the stereo shutter glasses worn by the subject (Crystal Eyes, StereoGraphics Inc.). The immersive virtual environment had no background textures and included only a cursor and a target (generated as 3D virtual ball-shaped targets with a 4.5 cm radius) in the field of view. Targets appeared one at a time at various locations and could be both seen (using VR technology) and felt (using robotics to render haptic sensation) (Figure 1A). Patients sat in a dark room on a chair placed in front of the VRROOM system, grasping the handle of the robot with their hand. The VRROOM is capable of recording events occurring in the scene (e.g., appearance of a target), as well as the subject’s arm and head position in space at any given time.

### Procedures

Patients were required to hold the handle of the robot and move the handle toward targets that appeared within the visuo-haptic virtual environment. Only one target and a cursor (representing the location of the tip of the robot) appeared within the scene at a time. Targets appeared randomly at various locations in the 3D space. Each trial continued until the target was acquired or 10 seconds elapsed. Following the completion of a trial, a new target appeared on screen. Patients were tested on 2 consecutive days. On each day patients completed 6 blocks of trials, each lasting 4 minutes and containing unlimited number of trials. That is, patients reached toward as many targets as possible. Patients were allowed to rest between blocks.

On each day, each pair of blocks included one of the following haptic conditions: (1) no haptic feedback (‘no force condition’), (2) a break-through force, similar to popping a balloon (‘break-through condition’), or (3) a gentle pulse of force (‘haptic nudge condition’). The order of the blocks presentation was randomized on both days.

The design of the break-through force is depicted in Figure 1B. In brief, forces were exerted in the following way: as patients approached the target, the robotic arm pushed back with a repulsor force. As they overcame the force, it gave way to an attractor force that pulled them smoothly to the center of the target. The attractor force was reduced to zero at the target center.

The haptic nudge was defined as a force of 1 N, lasting 250 ms, exerted in the direction of the target center. The nudge was applied if the system detected no movement (hand speed below 0.05 m/s) for a 1 s period. The nudge was intended to capture attention (that included a directional component) to cue patients to resume the movement.

### Data analysis

Spatial and temporal kinematic parameters of the reach movement were calculated for each trial. These included total trial time (s), from target appearance to trial completion, as well as hand path, velocity (m/s), and distance from target (m). The duration (s) of any pauses that occurred during the course of the movement were also calculated. A pause was defined as an interval of at least 50 ms in which the patient’s hand was not moving (velocity less than 0.06 m/s). The number of targets acquired in a block was also calculated. The Kolmogorov-Smirnov test was used to test for normality of the data. Statistical analysis was conducted using χ^2^ test (for number of pauses and nudges), paired t-test (for number of targets acquired), and one-way and two-way repeated measures ANOVAs with visit, force type (no force, break-through, or nudge), and block number as within-subject factors. The significance level was set at 0.05. Post-hoc analyses were conducted using Bonferroni tests.

## Results

### Tolerance of technology and procedure

Treatment during early stages of recovery in the TBI inpatient population is challenging; behavioral problems exhibited by some patients limit the ability to provide care. Therefore, it was important to first evaluate how such patients tolerate the technology and procedure. We have previously reported observations of subjective response to technology and procedure in a smaller group of patients [24]. Here we report that the interactive visuo-haptic environment was well-tolerated by 18 of the 21 patients, completing the experiments over two days. Three patients could not complete the 6 blocks in each visit due to either fatigue or excessive frustration. Patients exhibited no signs of intimidation by the technology and no deterioration in attention as the study progressed. Despite behavioral problems commonly exhibited in this population during therapy (e.g., agitation and restlessness), the interactive visuo-haptic environment was engaging and motivating for almost all patients. Using a problem behavior checklist adapted from the agitated behavior scale (see [24]) appropriate for characterizing patients at that low level of functioning, frequency of observed problem behaviors such as excessive talking, violent behavior, and removal of 3D glasses, has been documented in 15 patients and was reduced on the second visit.

During the study patients were exposed to forces delivered by the robot (i.e., nudge and break-through forces). We found that as the haptic forces came on, all patients tolerated them and were able to continue the experiment. Two patients responded to the haptic nudge by pulling in the opposite direction of the force. While one of these patients continued this response during both visits, this response diminished in the second patient as the study progressed.

### Attention loss during a task

**Figure 2:**
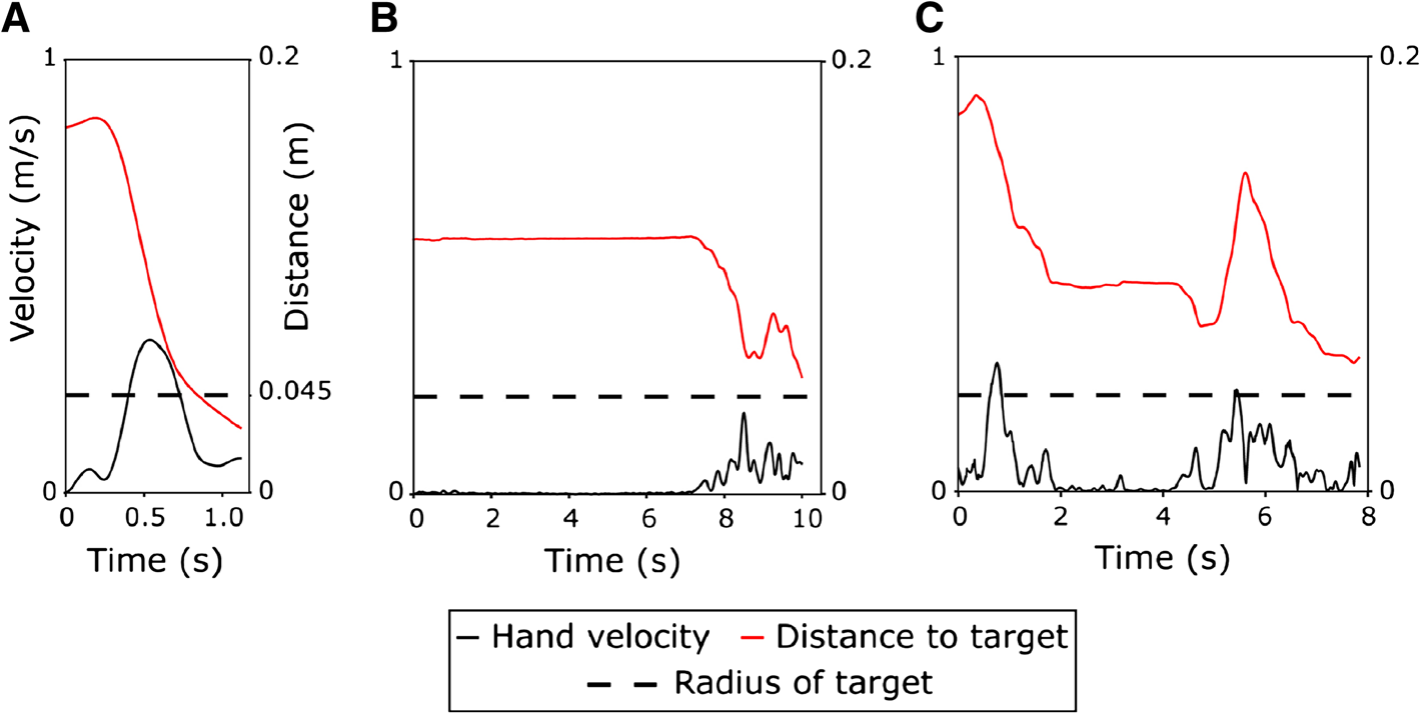
**Examples of hand velocity and distance from target from a representative patient. (A)** Ideal movement, **(B)** attention loss at movement initiation, **(C)** repeated attention loss during a movement (pauses during motion).

Attention loss during a task is common following TBI. We examined whether attention loss occurred between discrete movements or during a movement. Kinematic analysis of arm movements revealed attention loss both before and during a reaching movement. Investigation of the arm velocity and distance from target indicated that some movements contained prolonged movement initiation and/or a pause (or multiple pauses) occurring during the course of the movement (Figure 2). Whereas this was evident during both visits, interestingly, on the second visit patients exhibited significantly less pauses (χ^2^(1) = 261, p < .0001) and shorter pause duration (repeated measures ANOVA: F(1,17) = 9.4, p = 0.007).

### Effects of haptic feedback type on performance

Analysis of the effect of force type on performance (i.e., the number of targets acquired in a block of trials) was conducted for the three conditions (no force, break-through, and nudge). We first tested for normality of the “number of targets acquired” using the Kolmogorov-Smirnov test, which showed that data was normally distributed (p > 0.9). Table 2 summarizes the means and standard deviations for the measured variables for the three haptic conditions across the two visits.

**Table 2:** Summary of means and standard deviation for measured variables, for the three haptic conditions across visits

	**Visit 1**			**Visit 2**		
**Measured variables**	**No force**	**Break-through**	**Nudge**	**No force**	**Break-through**	**Nudge**
# Targets acquired	79.4 ± 46.7	78.7 ± 42.5	85 ± 44.1	107.9 ± 41.7	101.5 ± 43.8	108.4 ± 43.4
# Pause per trial	2 ± 2.4	2 ± 2.5	2.2 ± 3.3	1.4 ± 1.9	1.4 ± 1.8	1.5 ± 2.4
Pause duration (ms)	305.3 ± 525	299.6 ± 505.1	312.8 ± 630	295.8 ± 624.3	294.6 ± 644.2	246.7 ± 385.8
# Nudges per trial	-	-	2.46 ± 1.9	-	-	2.1 ± 1.6

Number of nudges per trial was calculated only for the trials where subjects received a nudge.

**Figure 3:**
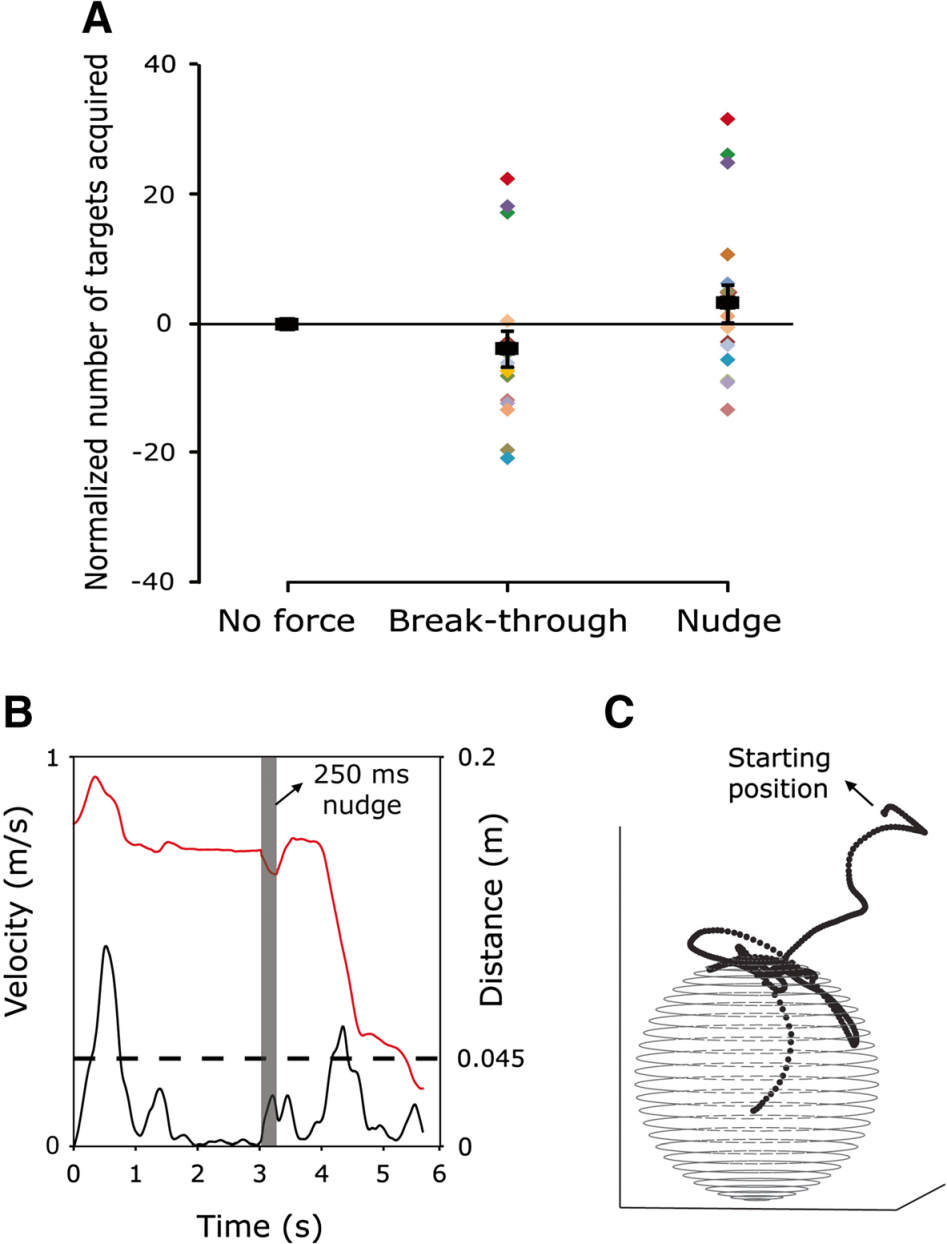
**Effects of haptic feedback type on performance. (A)** Normalized number of targets acquired per haptic feedback type. Mean (±SE) and individual subject data, collapsed across visits. **(B)** Hand velocity and distance from target from a representative patient during the haptic nudge condition. Patient completed the movement after receiving a haptic nudge. **(C)** Hand path from a representative patient during the break-through condition, showing how the cursor moved on the surface of the force boundary before penetrating the target.

Figure 3A shows the mean and individual subject data for each condition, collapsed across visits, calculated by subtracting the number of targets acquired from the no force condition. To investigate the effect of force type for each visit, a two-way repeated measures ANOVA (visit x force type) was conducted. A significant main effect of visit (F(1,17) = 437, p < 0.0001) was found, indicating that patients acquired more targets on the second visit. This held true for all three force conditions. In addition, a significant main effect of force type (F(2,34) = 16.4, p < 0.0001) was found, with no interaction. Bonferroni post-hoc test indicated that for the first visit, the break-through and no force were different than the nudge (p < 0.02), and resulted in less targets acquired compared to the nudge. Break-through and no force however were not different from each other. For the second visit, the nudge and no force were different than the break-through (p < 0.002), and resulted in more targets acquired compared to the break-through. Nudge and no force were not different from each other.

Results indicated benefits in most patients from the haptic nudge both before and during a movement. These patients regained concentration on the task and were able to complete the reaching movement and acquire the target (Figure 3B). The beneficial effect of the haptic nudges compared to the other two conditions however was obvious mostly on the first visit. This was in part related to the number of nudges provided, as patients needed less assistance from nudges as the study progressed. The overall number of nudges documented on the first and second visits (776 and 469 respectively) showed a significant decrease of 40% (χ^2^(1) = 75, p < .0001). Note, however, that although there was an overall beneficial effect of the haptic nudges, 5 of 21 individuals showed a slight performance decrement (Figure 3A).

Compared to nudge and no force, break-through resulted in less targets acquired on both visits. To further investigate why break-through forces somewhat degraded performance, we separated the total trial time (from target appearance to trial completion) into the time spent “outside” and “inside” of the force sphere. That is, the time from target appearance till the cursor reached the boundary representing a sphere around the target in which forces were active, and the time the cursor was on or within that force sphere. Since nudge and no force conditions included no such force boundary, the same sphere dimensions were used to calculate the time separation for these conditions. Repeated measures ANOVA collapsed across visits on the time spent “inside” the force sphere showed a significant main effect of force type (F(2,34) = 24.1, p < 0.0001), indicating longer time for break-through condition. Bonferroni post-hoc test showed that time on break-through trials was significantly longer than on the no force and nudge conditions (p < 0.0001) which were not different from each other. This held true on both the first and second visits.

An intriguing observation of the hand path revealed that in some patients the force boundary (in the break-through condition) was apparently perceived as an obstacle. Once the cursor reached the boundary, patients moved the cursor on the surface of the boundary before overcoming the force and moving toward the center of the target (Figure 3C). This resulted in prolonged time spent “inside” the force sphere.

### Improvement in performance

**Figure 4:**
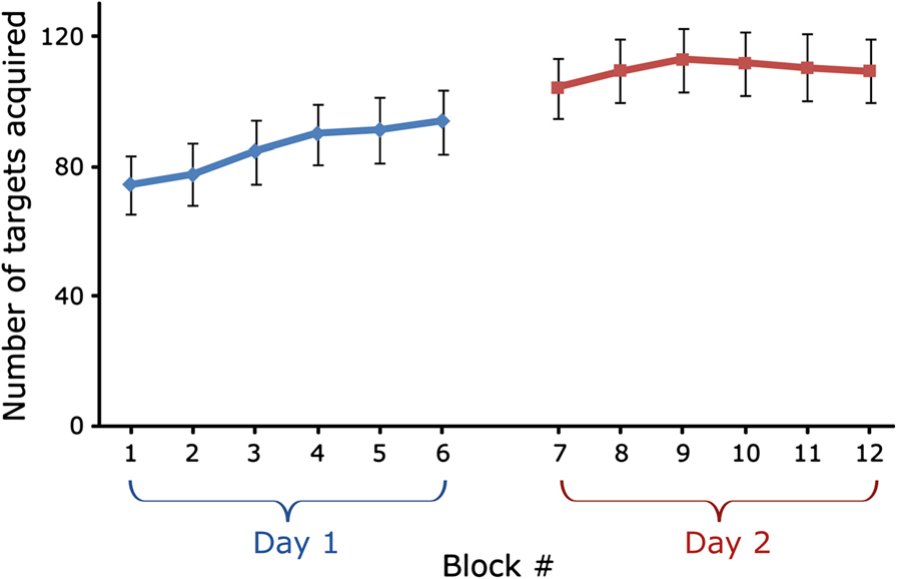
Mean (±SE) number of targets acquired across the 12 blocks of trials (6 blocks per visit).

Patients completed 6 blocks of trials each visit (total of 12 blocks). Analysis of the number of targets acquired as the study progressed (across the 12 blocks of trials) showed a clear improvement over the two visits (Figure 4). A two-way repeated measures ANOVA (visit x block number) revealed a significant increase in the number of targets acquired, suggesting that patients benefited from practice across blocks. Analysis revealed a main effect of visit (F(1,17) = 20.2, p = 0.0003), and a main effect of block number (F(5,85) = 8.95, p < 0.0001), but this was especially true for the first visit (F(5,85) = 10.28, p < 0.0001). Analysis also revealed an interaction (F(5,85) = 4.64, p = 0.0009). Bonferroni post-hoc test indicated that number of targets acquired was significantly lower for the first two blocks than for the other blocks (p < 0.005).

Interestingly, a paired t-test on the number of targets acquired in the last block on the first visit and in the first block on the second visit approached significance (t(17) = −2, p = 0.06). Although not significant (due to large variability in the data), there is an obvious trend suggesting that patients improved overnight between visits.

## Discussion

The current study tested a “virtually minimal” training paradigm for restoring attention in the severely impaired TBI inpatient population in the early stages of recovery. This study showed that such interactive visuo-haptic environments are well-tolerated (technology and procedure, including tolerance of forces), engaging, and promote improvement in this population (see also [24]). Subjects typically remained attentive to the task, improved progressively from session to session, and also showed spontaneous improvement from one day to the next. This approach could be beneficial for developing attention training technology for this inpatient population.

Only a few studies have explored treatment of the severely impaired inpatient TBI population [6,7]. Since patients often exhibit severe impairment of both focused and sustained attention, and since they are easily distracted by visual and auditory stimuli in the clinical environment they have great difficulty in staying engaged and sometimes withdraw from therapy or research. Evidence-based reviews support the effectiveness of attention rehabilitation following TBI in chronic and more high-functioning patients, but there is a lack of evidence to either support or recommend against attention training for the acute inpatient [4,17,18]. Our results agree with these authors in their recommendation that computer-based interventions can be an adjunct to clinician-guided treatment for rehabilitation of attention.

There are several reasons why methods that use interactive visuo-haptic environments have potential advantages over more standard forms of clinical care for the inpatient TBI population. The virtually minimal approach (a dark space with only a cursor and a target) eliminates extraneous distractions to a level not possible in a clinic environment, while allowing the clinicians to provide verbal cues and hand-on-hand assistance. Furthermore, targets are both seen and felt (haptically) by the subjects, engaging the sensory, tactile, and proprioceptive systems in the practice. Some subjects also perceived the task as a video game that drew and sustained their attention. Finally, the device can tirelessly and safely administer the task for prolonged periods with little or no supervision while measuring and recording accurate performance information on the subject.

Interestingly, patients in our study appeared to benefit from practice and to improve overnight between visits. Previous studies have shown that sleep contributes to memory consolidation [36]. Our findings were surprising since patients who are Rancho scale level V’s are expected to have very little memory retention from one day to the next, while Rancho IV’s typically have none. Since we observed both improvement and retention, we believe these approaches have potential for rehabilitation in this population. Further study is necessary to show a functional benefit that accumulates over multiple repeated visits during the inpatient’s stay.

Our observations showed that attention loss during a task was common and could occur either *between* (indicated by prolonged initiation or no initiation) or *during* movements. While failure to initiate is a well-known phenomenon in this population, it is unclear whether it is fully due to attention loss or to some other neurological mechanisms seen in other neuropathology such as Parkinson’s disease. Paused movements have been previously reported in healthy individuals and Parkinson’s disease patients [37,38]. These studies however required subjects to reach for targets in the presence of a visual perturbation (a target displacement and roll visual motion), which interfered with the ability to update the visuomotor response to the visual changes. Interestingly, the pauses we observed during motions are a striking result in the context of motor control theories, which suggest that limb actions involve a “ballistic” launch phase followed by corrections if necessary [39]. During reaching movements, the brain computes an estimate of future limb positions based on both predictive internal models and real-time feedback. The launch phase (before any feedback has yet arrived) appears to be completely interrupted on some patients’ motions, suggesting that this so-called ballistic phase may be much less automatic and uninterruptable than has been originally assumed. Such observed mid-movement pauses may also be associated with the neurological disorder of *task impersistence*, which is a failure to re-initiate activity without receiving immediate reward [40] and is associated with frontostriatal dysfunction [41]. The observed pauses during movements might be explained either by attentional or motor control deficits also sometimes observed in these patients. Interestingly, all patients enrolled exhibited some movements in which the patterns were perfectly consistent with reaching movements made by healthy individuals. We would expect that if there were any motor control deficits, we would have observed them consistently in all movement. We observed none of the typical motor deficits normally seen in TBI, stroke and spinal cord injury, such a spasticity, weakness, or contractures. By contrast, attention deficit commonly can fluctuate from one moment to the next, and verbal cues can rapidly and effectively redirect attention. We observed that patients often responded well to a cue (the “nudge”) to resume movement following a pause, more consistent with an attentional etiology. We therefore suspect that deficits observed were due to attentional and not motor control deficits, although involvement of motor control deficit cannot be entirely ruled out.

Previous studies have shown the beneficial effect of integrating visual and haptic feedback on performance [33]. Studies have further demonstrated strong crossmodal links in spatial attention between the two modalities, and how tactile cues are effective in capturing and directing overt visual attention [29,30]. The rationale behind the inclusion of the force conditions in our study was to use this form of multisensory integration to enhance performance. Our findings indicated that the haptic nudge condition resulted in superior performance compared to the no force and break-through conditions, although the effect was not large. Most patients (but not all) benefited from the nudge (a haptic cue), which directed them back to task, both before and during a movement toward the target. Patients regained concentration on the task and were able to complete the reaching movement. As patients’ performance improved, the assistance they required to stay on task and complete a movement was reduced. This was evident on the second visit from the number of nudges provided (40% less on the second visit), as well as from the reduced frequency of problem behaviors and need for instructions and cues; see also [24]. This however needs further investigation in a protracted study with repeated treatments to determine whether nudges are optimal only for the very initial stages of rehabilitation and not required thereafter.

Surprisingly, there have been very few (if any) attempts to engage the attention of patients in an interactive task that not only engages several sensory systems, but also reduces distractions. To our knowledge only one published work used haptic feedback for training on a single individual with a chronic TBI [23]. This study showed results similar to ours -- the treatment was well tolerated and performance incrementally improved across practice. Engaging the nervous system with tactile feedback that is salient and highly relevant to the task may be a prominent method for engaging and sustaining the attention of these patients. However, because one mode of haptic feedback used in our study (i.e., break-through) increased completion times, it may be that some forms of tactile stimulation may also distract the patient and hence should be used with caution as well as evaluated for their efficacy. Interestingly however, these patients were still attending to the task and continued to move while interacting with the force boundary. Hence, while subjects might have switched to a new task -- exploring the surface, they appeared to remain tolerant of the procedure conditions and continued to attend to what they were doing.

The “virtually minimal” training approach used in this study included simple repetitive reaching toward targets over two visits. Patients’ performance improved as the study progressed, but leveled off during the second visit. This might reflect a “ceiling effect” of their performance. Furthermore, several patients mentioned that they were getting bored performing the same task in later sessions. Our minimal interactive environment approach allows for controlling the presented stimuli, environmental distractions, and level of assistance provided during the task. This foundational work also allows for further design of more progressive and challenging levels of interactive environments. While our approach appears to be most effective in the first few treatment sessions during early rehabilitation of TBI, a protracted study with repeated treatments that progressively challenges attention may be optimal. We have recently reported on a single case study that demonstrated how 2-week treatment approach was associated with improved engagement in therapy, decreased disruptive behavior, and more sensitive measurement of progress [42]. What is clear in the current preliminary work is the promise of such treatments for remediation of attention in this severely impaired population.

### Consent

Written informed consent was obtained from the subject for the publication of this report and the accompanying image.

## Competing interests

The authors declare that they have no competing interests.

## Authors’ contributions

AYD designed and conducted the experiment, performed the analysis and wrote the manuscript. MR conducted the experiment and performed the analysis. EBL and FSZ contributed to conception and design of the study and the interpretation of data. NH and SP conducted the experiment and made contribution for the interpretation of data. AS performed the analysis and was involved in revising the manuscript. JLP made contribution to conception and design of the study, interpretation of data, and revising the manuscript. All authors read and approved the final manuscript.
